# First Record of Invasive Lionfish (*Pterois volitans*) for the Brazilian Coast

**DOI:** 10.1371/journal.pone.0123002

**Published:** 2015-04-22

**Authors:** Carlos E. L. Ferreira, Osmar J. Luiz, Sergio R. Floeter, Marcos B. Lucena, Moysés C. Barbosa, Claudia R. Rocha, Luiz A. Rocha

**Affiliations:** 1 Departamento de Biologia Marinha, Universidade Federal Fluminense, Niterói, RJ, Brazil; 2 Department of Biological Sciences, Macquarie University, Sydney, Australia; 3 Departamento de Ecologia e Zoologia, Universidade Federal de Santa Catarina, Florianópolis, SC, Brazil; 4 California Academy of Sciences, San Francisco, CA, United States of America; 5 Department of Ecology and Evolutionary Biology, University of California Santa Cruz, Santa Cruz, CA, United States of America; California Polytechnic State University, UNITED STATES

## Abstract

The invasion of the northwestern Atlantic by the Indo-Pacific lionfish has developed extraordinarily fast, and is expected to cause one of the most negative ecological impacts among all marine invasions. In less than 30 years, lionfish have dramatically expanded their distribution range to an area encompassing the eastern coast of the USA, Bermuda, the entire Caribbean region and the Gulf of Mexico. The rapidity of the lionfish spread has raised concerns in other parts of the Atlantic that may be under the reach of the invasion. Despite the anticipation that lionfish would eventually extend their range throughout most of the eastern coast of South America, it had not been recorded in Brazil until now. Here we report the first lionfish appearance for the Brazilian coast and show that the individual collected by us is genetically linked to the invasive Caribbean population. Since small-range endemics are found in several locations in Brazil and are among the species that are most vulnerable to extinction, we recommend urgent control, management and education measures aimed at minimizing the effects of this impending invasion.

## Introduction

The invasion of the northwestern Atlantic by the Indo-Pacific lionfishes, *Pterois volitans* and *P*. *miles* (hereafter referred to as lionfish), has developed extraordinarily fast [1,2], and is expected to cause one of the most negative ecological impacts among all marine invasions [3]. In less than 30 years, lionfish have dramatically expanded their non-native distribution range to an area of roughly 7.3 million km^2^, encompassing the eastern coast of the USA, Bermuda, the entire Caribbean region and the Gulf of Mexico [1,4].

Despite being an iconic representative of the coral reef fauna, lionfish are habitat generalists also found in mangroves, seagrasses, mud bottoms, mesophotic reefs and even estuaries [5–7], surviving well in low salinity waters [8]. Therefore, their expansion should not be constrained by the Amazon-Orinoco Plume (AOP), a huge freshwater and sediment discharge of the Amazon and Orinoco Rivers that represents a substantial barrier for reef-associated organisms’ dispersal between the Caribbean and the coast of Brazil [9,10]. Here we report the first lionfish appearance in the Brazilian coast and show results of a genetic analysis of this first individual.

## Methods

**Fig 1:**
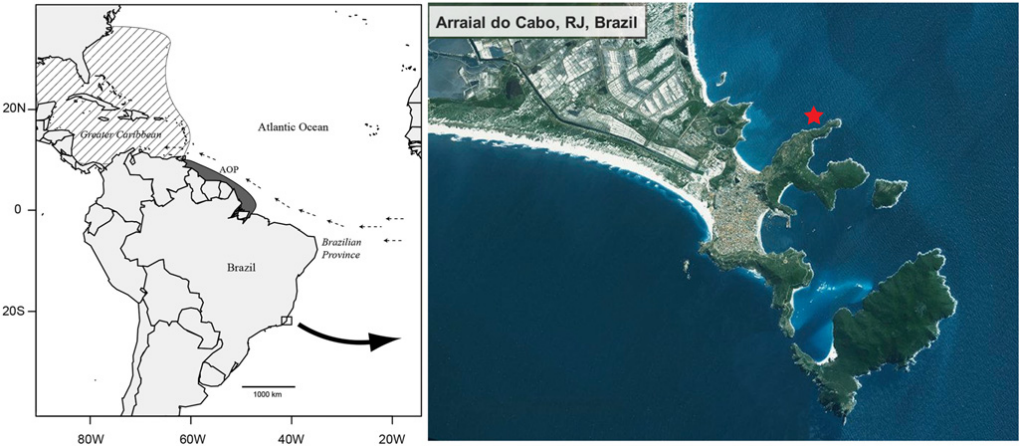
Location (marked with a red star) where the Lionfish was collected in southeastern Brazil. Satellite image by NOAA (http://maps.ngdc.noaa.gov/viewers/bathymetry/).

**Fig 2:**
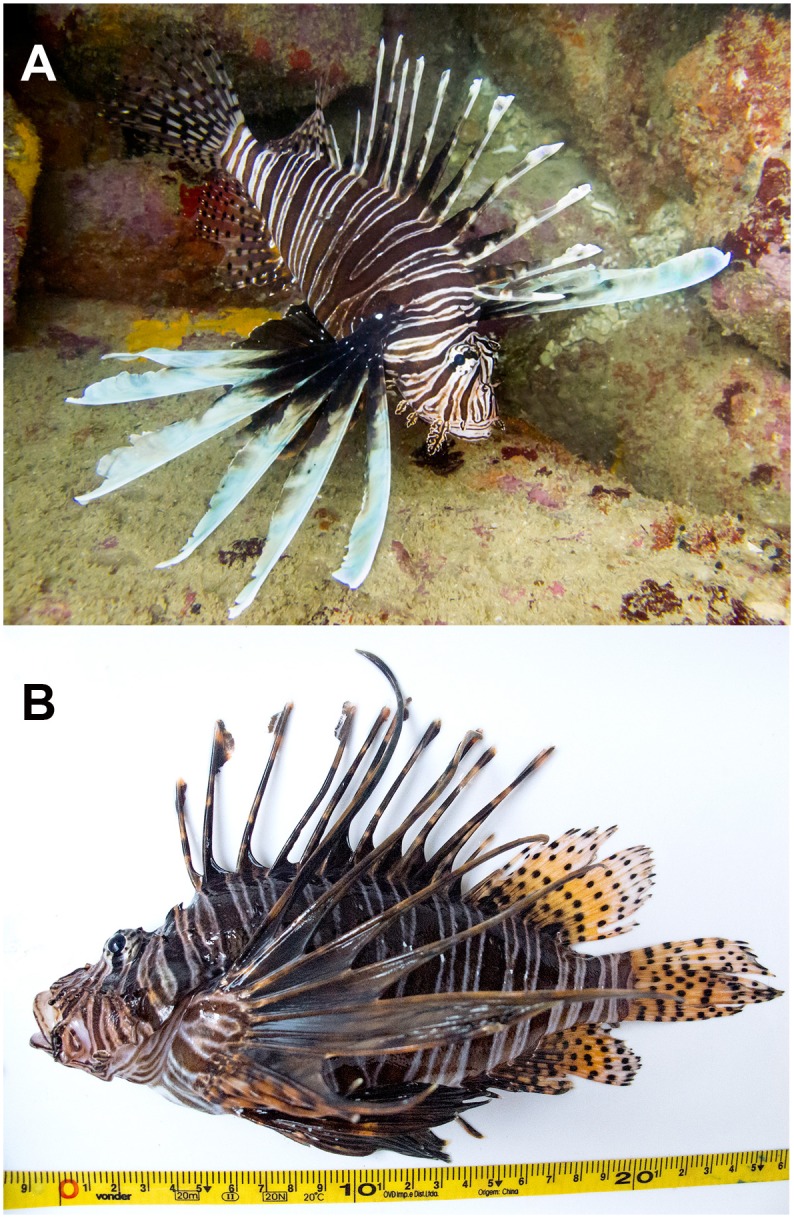
Underwater (A) and specimen photograph (B) of the Lionfish collected in Arraial do Cabo, SE Brazil. Photos by MBL and MCB.

The first lionfish found in the Brazilian coast was spotted by a group of recreational divers on May 10, 2014, in the rocky shores of Arraial do Cabo, in the southeastern coast of Brazil (22°57'8.69"S, 42° 0'32.71"W, Fig 1). It was an adult specimen (25 cm TL; Fig 2), which remained on the same patch reef for at least two days until it was collected using a hand spear and identified morphologically as *Pterois volitans*. Tissue samples from gill and muscle were collected and preserved in ethanol. Stomach contents were also preserved in ethanol. The specimen was fixed in formalin, preserved in ethanol and vouchered at the ichthyology collection of the Universidade Federal do Espírito Santo (CIUFES 3252). In order to elucidate the provenance of the Brazilian lionfish, we sequenced two mitochondrial DNA genes: cyt *b* (cytochrome b) and COI (cytochrome oxidase I). Methodology for sequencing followed standard techniques described in detail by Hamner et al. [11] for cyt *b* and Valdez-Moreno et al. [12] for COI.

## Results and Discussion

Because of strong founder effects, the Caribbean lionfish population has a unique genetic signature that differs from their Indo-Pacific native counterparts [2,11,12]. Therefore, if the genetic signature of the lionfish captured in Brazil matches Indo-Pacific individuals, we would assume that the individual did not originate from populations in the Caribbean. However, the DNA sequences from the Brazilian lionfish matched Caribbean individuals of *Pterois volitans*: the 810 base pairs cyt *b* sequence (Genbank accession no. KP641131) that we obtained is 100% identical to haplotype C, the second most common haplotype in the US Atlantic coast [11], and the 652 base pairs COI sequence (Genbank accession no. KP641132) from our fish is 100% identical to haplotype B, the most common haplotype in Mexico [12].

The lionfish invasion of the Brazilian coast was expected to start closer to the Amazon mouth, in either the north or the northeast region of Brazil. Our finding at Arraial do Cabo, a subtropical reef ~5,500 km away from the Caribbean, is surprising and raises the question of whether this individual is the outcome of a long-distance larval dispersal event from a Caribbean source or a secondary release from the aquarium trade. Even though we cannot rule out the latter hypothesis, two observations make it unlikely: first, export data from the US (where Brazil gets most of its imported marine fish) indicate that Caribbean lionfish are very rarely exported (R. Talbot pers. comm.); second, the lionfish analyzed here was collected in Cabo Frio, a sparsely populated area with no major aquarium stores and very few hobbyists. In addition, the lack of records in northeastern Brazil may also be explained by the relatively lower frequency of diving in that region. Therefore, it is our opinion that the lionfish recorded here arrived in Brazil via natural larval dispersal from the Caribbean.

In Brazil, lower species richness, high endemism and consequent lower functional redundancy of reef fish assemblages [13] suggest that a potential lionfish invasion will have even direr consequences than in the Caribbean reefs. Historically, the country lacks monitoring programs aimed at early detection of marine invasive species. Furthermore, current regulations concerning aquarium trade of exotic species do not contemplate potential of invasiveness [14]. Since lionfish control efforts seem to be working in many places in the Caribbean [15–17], we suggest the implementation of a monitoring program that includes seldom visited areas in the northeastern region and immediate population control actions through removal in as many areas along the Brazilian coast as possible. This initiative should be embraced by one of the Brazilian Conservation National Action Plans (PAN) programs in development. Lionfish sightings should also be reported to the US Geological Survey, which maintains a comprehensive database (http://nas.er.usgs.gov/sightingreport.aspx). Finally, a strong educational program raising awareness about the dangers of lionfish and other marine invasive species aimed at aquarium shops, aquarium hobbyists and SCUBA divers should be started as soon as possible.
